# Olmesartan Attenuates the Impairment of Endothelial Cells Induced by Oxidized Low Density Lipoprotein through Downregulating Expression of LOX-1

**DOI:** 10.3390/ijms13021512

**Published:** 2012-02-01

**Authors:** Hua Zhang, Genshan Ma, Yuyu Yao, Huidong Qian, Weizhang Li, Xinjun Chen, Wenlong Jiang, Ruolong Zheng

**Affiliations:** 1 Department of Cardiology, Jiangsu Jiangyin People’s Hospital, 163 Shoushan Road, Jiangyin, Jiangsu, 214400, China; E-Mails: zhang3478@163.com (H.Z.); jaisdfj @yeah.net (H.Q.); dafasdfas@126.com (W.L.); yuji875@163.com (X.C.); jaing3@163.com (W.J.); 2 Department of Cardiology, Zhongda Hospital of Southeast University, 87 Dingjiqiao Hunan Road, Nanjing, Jiangsu, 210009, China; E-Mails: eraervf@163.com (G.M.); zheifrek @163.com (Y.Y.)

**Keywords:** olmesartan, lox-1 receptor, endothelial cells, oxidized low density lipoprotein, apoptosis

## Abstract

Oxidized low density lipoprotein (ox-LDL) and its receptor, lectin-Like ox-LDL receptor-1 (LOX-1), play important roles in the development of endothelial injuries. Olmesartan can protect endothelial cells from the impairment caused by various pathological stimulations. In the present study we investigated whether olmesartan decreased the impairment of endothelial cells induced by ox-LDL by exerting its effects on LOX-1 both *in vitro* and *in vivo*. Incubation of cultured endothelial cells of neonatal rats with ox-LDL for 24 h or infusion of ox-LDL in mice for 3 weeks led to the remarkable impairment of endothelial cells, including increased lactate dehydrogenase synthesis, phosphorylation of p38 mitogen-activated protein kinases (p38 MAPK) and expression of apoptotic genes such as B-cell leukemia/lymphoma 2 (Bcl-2)-associated X protein (Bax) and caspase-3. Simultaneously, the cell vitality and expression of Bcl-2 gene were greatly reduced. All these effects, however, were significantly suppressed by the treatment with olmesartan. Furthermore, ox-LDL promoted up-regulation of LOX-1 expression either in cultured endothelial cells or in the aortas of mice, which was reversed with the administration of olmesartan. Our data indicated that olmesartan may attenuate the impairment of endothelial cell via down-regulation of the increased LOX-1 expression induced by ox-LDL.

## 1. Introduction

Endothelial cell injury not only leads to endothelial dysfunction, but also is an independent risk factor for atherosclerosis [1]. It is therefore essential to prevent and suppress the development of endothelial cell injuries caused by various pathological factors. Previous studies have shown that oxidized low density lipoprotein (ox-LDL) and its receptor, lectin-Like ox-LDL receptor-1 (LOX-1), were crucial in the response to endothelial cell insults [2–4]. Studies using primary cultured endothelial cells have demonstrated that ox-LDL could directly induce apoptotic gene expressions and consequent cell apoptosis [2]. In human coronary artery endothelial cells, ox-LDL was found to promote cell growth via LOX-1-mediated angiotensin converting enzyme expression [5]. Continuous exposure to ox-LDL in rats resulted in an increased number of injured endothelial cells and resultant injury to the endothelium, independent of its pressure-elevating effect [6]. Anti-LOX-1 antibody significantly suppressed endothelial cell injury in the absence of hypertension [4,7,8]. These results clearly indicated that ox-LDL and LOX-1 play critical roles in the development of endothelial cell injury and atherosclerosis. Clinically, the statin drugs have been generally and effectively used in the treatment of atherosclerosis elicited by endothelial injury. However, accumulating evidence suggests that angiotensin II receptor blocker also has protective effects on endothelial cell injuries caused by a variety of pathological stimulations. However, the underlying mechanisms remain to be elucidated.

Olmesartan, clinically used as an angiotensin II receptor blocker, plays a critical role in preventing cardiac hypertrophy by exerting its inhibitory effects on the angiotensin II receptor which is an important component of the renin-angiotensin system (RAS) [9]. Olmesartan may also have anti-oxidant and anti-inflammatory effects on patients with heart failure [10]. Clinical statistics indicated that the ox-LDL levels are independent risk factors of atherosclerosis [11]. Also, a cross-talk between RAS and clusters of lipoproteins has been suggested [12–15]. Olmesartan may reduce H_2_O_2_-induced upregulation of LOX-1 expression in human aortic endothelial cells [16]. In the present study we investigated whether olmesartan decreased the impairment of endothelial cells induced by ox-LDL by exerting its effects on LOX-1 both *in vitro* and *in vivo.* Our findings suggested that olmesartan might play a role in the inhibition of endothelial cell injuries induced by ox-LDL and thereafter in the suppression of atherosclerosis through down regulation of LOX-1 expression.

## 2. Results and Discussion

### 2.1. Olmesartan Inhibited Apoptotic Responses Induced by ox-LDL in Cultured Endothelial Cells *in Vitro*

To determine the roles of olmesartan in endothelial cell injuries during ox-LDL exposure, we measured the cellular vitality, nitrogen monoxide (NO) and Lactate Dehydrogenase (LDH) synthesis. Our results showed ox-LDL significantly increased the LDH synthesis and decreased cell vitality and NO synthesis in neonatal rat endothelial cells (Table 1).

**Figure 1:**
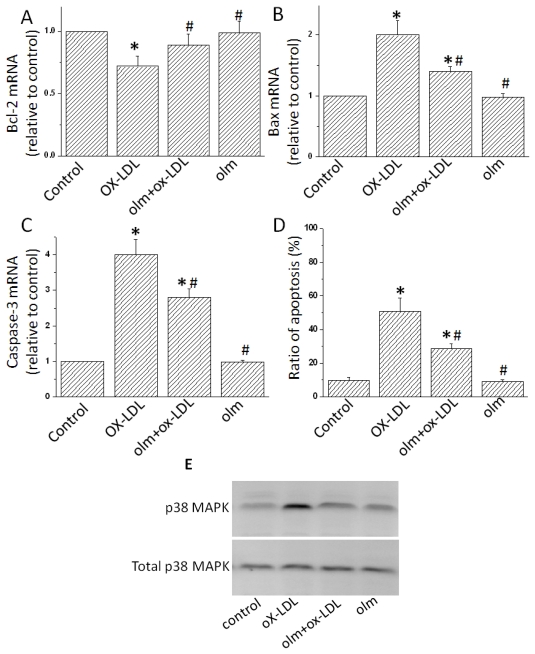
Effects of olmesartan on Oxidized low density lipoprotein (ox-LDL)-induced endothelial cell injuries in cultured endothelial cells. (**A–D**) Cultured endothelial cells of neonatal rats were added by vehicle (Control), ox-LDL (200 μg/mL), olmesartan (10^−5^ M) or olmesartan (10^−5^ M) plus ox-LDL (200 μg/mL) for 24 h. Expressions of Bax, Caspase-3 and Bcl-2 genes were evaluated by Real-time Polymerase chain reaction (PCR). Glyceraldehyde 3-phosphate dehydrogenase (GAPDH) was served as the internal control. (**E**) Phosphorylation of p38 Mitogen-activated protein kinase (MAPK). P38 MAPK was examined by Western Blot using an anti-phosphor-p38 MAPK antibody. Total p38 MAPK were used as the loading control. Representative photograms from three independent experiments are shown. All data are expressed as mean ± S.E.M from three independent experiments. *p < 0.01 *vs.* endothelial cells in Control; ^#^ *p* < 0.05 *vs.* endothelial cells treated with ox-LDL. Olm, Olmesartan; ox-LDL, Oxidized low density lipoprotein; LDL, low density lipoprotein. The same as the other figures.

We then determined whether ox-LDL induced cellular apoptosis. Treatment with ox-LDL enhanced the apoptosis of endothelial cells by approximately 5 fold (Figure 1D). The up-regulation of phosphorylation of p38 MAPK was thought to be an important response to some stimulation of endothelial cells [17]. We therefore examined the phosphorylation levels of p38 MAPK in ox-LDL-treated endothelial cells. The phosphorylation levels were significantly increased by ox-LDL treatment (Figure 1E). Meanwhile, we also found an increased expression of apoptotic genes, including Bax and Caspase-3, while a decrease in the Bcl-2 (Figure 1A–C). These findings were consistent with previous research [4]. The addition of olmesartan to ox-LDL-stimulated endothelial cells significantly alleviated all the above harmful effects, including decreased cell vitality, increased LDH secretion, upregulated p-38 MAPK and overexpression of apoptotic genes such as Caspase-3 and Bax (Figure 1). These results indicated anti-apoptotic effects of olmesartan on endothelial cell injuries induced by ox-LDL *in vitro*.

### 2.2. Olmesartan Inhibited ox-LDL-Induced Endothelial Cell Injury *in Vivo*

**Figure 2:**
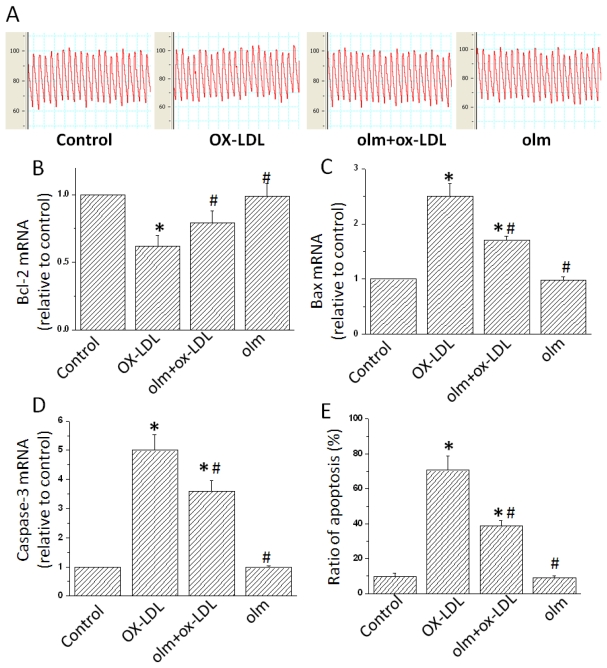
Inhibition of Oxidized low density lipoprotein (ox-LDL)-induced endothelial cell injuries by olmesartan in mice. C57BL/6 mice were infused with vehicle (Control), ox-LDL (8.4 mg/kg/day), olmesartan (3 mg/kg/day) or ox-LDL (8.4 mg/kg/day) plus olmesartan (3 mg/kg/day) for 3 weeks. (**A**) Blood pressure (BP) recordings. Representative recordings from five mice are shown. (**B–E**) Expressions of Bax, Caspase-3 and Bcl-2 genes evaluated by Real-time Polymerase chain reaction (RT-PCR). Glyceraldehyde 3-phosphate dehydrogenase (GAPDH) were used as the internal control. All values are expressed as mean±S.E.M. from five mice in all the groups. * *p* < 0.05 vs. endothelial cells in Control; ^#^ *p* < 0.05 vs. endothelial cells treated with ox-LDL.

To determine the roles of olmesartan against ox-LDL insults in endothelial cells *in vivo*, we treated the mice with ox-LDL (8.4 mg/kg/day), olmesartan (3 mg/kg/day) or both of them. Of course, the dose of ox-LDL at 8.4 mg/kg/day was insufficient to elevate the BP of mice. Three weeks later, we found that BP was not significantly different among all groups of mice (Figure 2A). However, Western Blotting analysis showed endothelial cells from the aortas of mice were significantly impaired in ox-LDL-infused mice, including increased cellular apoptosis (Figure 2E). Also, in the aorta vessels in mice treated with ox-LDL, expressions of Bax and Caspase-3 were up-regulated while Bcl-2 was suppressed. These results were consistent with our findings of *in vitro* studies (Figure 2B–D). Interestingly, olmesartan significantly blunted the cellular injuries in terms of cell viability during the exposure of ox-LDL *in vivo* (Figure 2B–E). Moreover, treatment with olmesartan significantly decreased the gene expressions of Bax and Caspase-3 while increasing Bcl-2 levels in ox-LDL-treated mice. These results suggested olmesartan could protect endothelial cells against the injuries induced by ox-LDL *in vivo*.

### 2.3. Olmesartan Inhibited the Expression of LOX-1 in Ox-LDL-Treated Endothelial Cells

**Figure 3:**
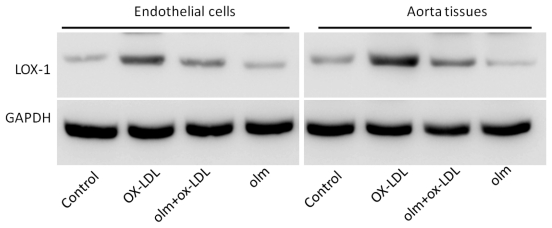
Effects of olmesartan on the expression of lectin-Like ox-LDL receptor-1 (LOX-1) protein. Cultured endothelial cells and mice were treated with vehicle, Oxidized low density lipoprotein (ox-LDL), olmesartan or ox-LDL plus olmesartan. Membrane proteins were extracted from endothelial cells and the aorta tissues of mice and subjected to Western Blot analyses for expression of LOX-1 protein using an anti-LOX-1 antibody. Glyceraldehyde 3-phosphate dehydrogenase (GAPDH) in whole cell lysate was used as the loading control. Representative photograms from three independent experiments for endothelial cells or from five hearts of mice are shown.

LOX-1 has been implicated in the development of endothelial cell injuries induced by ox-LDL [5]. Hence we tested whether olmesartan attenuated the ox-LDL-induced endothelial cell injuries by exerting its effects on LOX-1 expression. We detected the expression of LOX-1 in cultured endothelial cells and the aorta tissues of mice treated by ox-LDL. Ox-LDL treatment significantly induced up-regulation of LOX-1 expression both in the cultured endothelial cells and the aorta tissues, and olmesartan significantly reduced the increased LOX-1 expression elicited by ox-LDL, suggesting that olmesartan may exert its anti-apoptotic effects by down-regulation of LOX-1 (Figure 3).

## 3. Experimental Section

### 3.1. Animal Models

C57BL/6 mice and neonatal Sprague Dawley rats were purchased from Shanghai Laboratory Animal Center, Chinese Academy (Shanghai, China). Ox-LDL (8.4 mg/kg/day, Sigma-Aldrich, St. Louis, MO, USA), vehicle or olmesartan (3 mg/kg/day, Santa Crus, Santa Cruz, CA, USA) was continuously administered using Alzet micro osmotic minipumps (Model 2002, Durect, Cupertino, CA, USA) implanted subcutaneously into the mice. All protocols were approved by the Animal Care and Use Committee of Dongnan University and in compliance with “Guidelines for the Care and Use of Laboratory Animals” published by the National Academy Press (NIH Publication No. 85–23, Revised 1996).

### 3.2. Haemodynamic Measurements

Blood pressure (BP) was evaluated as previously described [18]. A micronanometer catheter (Millar 1.4F, SPR 835, Millar Instruments, Inc. Houston, TX, USA) was inserted into the right common carotid artery. The transducer was connected to Power Laboratory System (AD Instruments, Castle Hill, Sydney, Australia) and BP was recorded.

### 3.3. Cell Culture and Treatment

Neonatal rat endothelial cells were cultured in Dulbecco’s modified Eagle’s medium (DMEM) with 10% fetal bovine serum (FBS) for 24–36 h incubation depending on the status of the cells. The cells were then transferred to serum-free DMEM for 24 h. Ox-LDL (200 μg/mL), olmesartan (10^−5^ M) or vehicle was added to the cells. After 24 h incubation all the endothelial cells were collected and lysed for the further analysis.

### 3.4. Cell Viability Assay and Nitric Oxide (NO) Production Assay

Endothelial cells were plated in 96-well cell culture plates. When the cultured cells became sub-confluent, they were washed once with DMEM medium. Cell viability was determined by 3-(4,5)- dimethylthiahiazo(-z-y1)-3,5-di-phenytetrazoliumromide (MTT) assay, as described previously [19]. The viability was calculated as: percent = (*OD* of treated group/*OD* of control group) ×100. For NO production assay, the cells were treated as described above. Then 100 μL of culture solution was used for the NO (Nitric Oxide) assay. NO formation was detected with a NO Detection Kit following the manufacturer’s protocol (Intron Biotechnology, Korean). The kit accurately detects the concentration of NO- by indirectly measuring nitrite. The optical density was measured at a wavelength of 550 nm. Lactate dehydrogenase (LDH) assay was performed on the cells using an LDH kit according to the manufacturer’s protocol (Cayman Chemical Company, Ann Arbor, MI, USA). Light absorption was measured with a Cintra 5 UV-vis spectrometer (GBC, Sydney, Australia).

### 3.5. Detection of Vascular Endothelial Cell Apoptosis Using Flow Cytometry

For the sample preparation *in vivo*, the arteries of mice were rinsed with phosphate buffered saline containing 100 U/mL penicillin and 100 μg/mL streptomycin. Vascular endothelial cells were isolated by 0.25% (W/V) trypsin digestion method. The digestion lasted for 25 minutes. DMEM with 10% FBS was used to terminate the reaction, and phosphate buffered saline was used to rinse the sample. The digestion fluid was centrifuged to collect endothelial cells for the detection of the apoptosis ratio. *In vitro*, cells were collected after treatment and rinsed in phosphate buffered saline three times. The cells were centrifuged at 450 *g* for 5 minutes and the supernatant liquid was removed. The cell suspension was then fixed in pre-cooled 70% (V/V) ethanol at 4 °C for 24 h. The cell density was adjusted to 1 × 10^5^ cells/mL. The fixed cells were treated with propidium iodide staining solution (100 μg/mL) for 30 minutes in the dark and apoptosis was measured using a flow cytometer (Beckman. Carlsbad, CA, USA). The percentage of apoptotic cells was calculated as follows: percentage of apoptotic cells = (cell number at sub-double peak/total cell number) × 100%.

### 3.6. Real-Time RT-PCR

Total RNA was isolated from the rat vascular tissues or cultured cells using TRIZol reagent according to the manufacturer’s instructions. After purification RNA was subjected to the Real-time RT-PCR analysis for gene expressions of B-cell leukemia/lymphoma 2 (Bcl-2), BCL2-associated X protein (Bax) and caspase-3 on a BIO-RAD IQ5 multicolor detection system using the respective primers (Table 2). Melting curves and quantization were analyzed using IQ5 multicolor detection system software, respectively. A comparative CT method was used to determine relative quantification of RNA expression. The gene expression of Glyceraldehyde-3-phosphate dehydrogenase (GAPDH) was used as the internal control. All PCR experiments were repeated at least in triplicate.

### 3.7. Western Blot Analyses

Total proteins were extracted from mouse aorta tissues or culture cells and analyzed by Western blot to determine the levels of phosphorylation of p38 MAPK. The amount of LOX-1 was examined after isolating the membraneous and cytosolic fractions from total proteins. Briefly, cells were lysed and first centrifuged at 200 *g* to remove nuclei. The supernatant liquid was then centrifuged at 15,000 *g* for 30 min to pellet the cell membrane. The total proteins or pelleted membranes were size-fractionated by SDS–PAGE and transferred to Immobilon-P membranes (Millipore, Billerica, MA, USA). The blotted membranes were incubated with antibodies against phosphorylated p38 MAPK (Cell Signaling Technology, Beverly, MA) and LOX-1 (Santa Cruz Biotechnology, Santa Cruz, CA, USA), and subjected to an ECL Detection system (GE Healthcare, Piscataway, NJ, USA). GAPDH was used as the internal control.

### 3.8. Statistical Analysis

Data are Mean ± S.E.M. Comparison was performed by one-way or two-way analysis of variance followed by Newman–Keuls test for post hoc analysis to determine the difference among groups.

## 4. Conclusions

Previous studies indicated that the protective role of olmesartan against cardiovascular disorders was largely attributed to its key effects in anti-hypertension [20]. However, a variety of evidence has revealed that olmesartan could also be involved in other biological activities, such as anti-inflammation, anti-oxidation and anti-apoptosis independently of anti-hypertension [21–23]. Such properties of olmesartan could ameliorate a series of cardiac events. Recent studies have shown that the underlying mechanisms that olmesartan has with the above effects may be to inhibit inflammatory neutrophil recruitment and endothelial cell apoptosis [24]. The present study also indicated that administration of olmesartan could inhibit endothelial cell apoptosis induced by ox-LDL both *in vitro* and *in vivo*. Furthermore, our results also suggested that the protective effects of olmesartan could be, at least in part, due to inhibition of LOX-1 up-expression induced by ox-LDL in endothelial cells.

Ox-LDL plays a critical role in cardiac growth, resulting in an increase in cardiac fibrosis, remodeling and endothelial dysfunction [25,26]. The present study provides evidence that olmesartan plays a protective role against ox-LDL-induced endothelial cell injuries. As our results have shown, endothelial cell ratio of apoptosis, release of LDH, and Bax and Caspase-3 mRNA expressions in the endothelial cells treated by ox-LDL *in vivo* were significantly increased in mice independently of BP. However, administration of olmesartan significantly attenuated all the injury responses in endothelial cells treated by ox-LDL. Also, our *in vitro* results showed similar findings. Taken together, our present study demonstrates that olmesartan could prevent endothelial cells from ox-LDL-induced injuries.

Our study reveals the suppression of LOX-1 expression by olmesartan, suggesting a potential involvement of the LOX-1 pathway. We thus postulate that the reduction of LOX-1 expression by olmesartan is a significant mechanism for subsequent reduced endothelial cell injury during ox-LDL stimulation. In the presence of cellular insults, the local RAS is activated [27]. Moreover, Li D. *et al.* found NF-kappaB activation seems to play a critical role in the regulation of AngII receptors, which provide a basis for the use of antioxidants and AT1R blockers in designing therapy of atherosclerosis [28]. Given the known protective effects of olmesartan, we hypothesize that olmesartan could also regulate LOX-1 under the conditions of endothelial cell injuries. To investigate this hypothesis, we administered olmesartan via continuous infusion of olmesartan into mice or addition of olmesartan to the cultured endothelial cells in the presence of ox-LDL stimulation. Since the biological efficacy of ox-LDL was determined by the expression levels of LOX-1, olmesartan-mediated down-regulation of LOX-1 could thus reflect its antagonistic effects against ox-LDL-mediated endothelial cell injuries. Here, the olmesartan-induced down-regulation of LOX-1 in endothelial cells was accompanied by a decrease of endothelial cell injuries. These findings were consistent with the recent report that olmesartan inhibited the nuclear accumulation of NF-kappa B [29], a downstream signal of ox-LDL-mediated LOX-1 activation. However, a detailed mechanism requires further studies. In conclusion, the present study demonstrates that olmesartan can protect endothelial cells from the injuries induced by ox-LDL, possibly via down-regulation of LOX-1 expression. Our results suggest a potential therapeutic strategy for the treatment of endothelial cell injuries by inhibition of LOX-1 levels.

## Figures and Tables

**Table 1:** Effects of olmesartan on Oxidized low density lipoprotein (ox-LDL)-induced endothelial cell injuries in cultured endothelial cells.

group	Cell vitality	LDH (U/L)	NO (μM)
Control	100%	40.2 ± 10.6	10.5 ± 0.9
ox-LDL	54.6% ± 9.2% *	86.5 ± 20.4 *	5.3 ± 1.2 *
ox-LDL+olmesartan	78.1% ± 6.2% *#	68.6 ± 13.7 *#	7.4 ± 2.5 *#
olmesartan	99.8% ± 1.2% #	38.7 ± 10.2 #	11.2 ± 1.8 #

Cultured endothelial cells of neonatal rats were added by vehicle (Control), ox-LDL (200 μg/mL), olmesartan (10^−5^ M) or olmesartan plus ox-LDL for 24 h.

* *p* < 0.05 *vs.* endothelial cells in Control;

# *p* < 0.05 *vs.* endothelial cells treated with ox-LDL.

NO: nitrogen monoxide; LDH: Lactate Dehydrogenase; 1 Unit (U) is the amount of LDH that catalyzes the reaction of 1 μmol of substrate per minute, U/L, total LDH activity per 1 liter sample; μM, micro mole per liter.

**Table 2:** Primer sequences.

Gene	Forward primer (5′-3′)	Reverse primer (5′-3′)	Size (bp)
Bax	Mouse: TCAGAACCATCATGGGCTGG	CTTCCAGATGGTGAGCGAGG	171
	Rat: GCTGATGGCA ACTTCAACTG	CGCTCACGGAGGAAGTCCAG	140

Bcl-2	Mouse: GCCAGTGTTCCATGCACCAA	CAAGTGGGAAGGTACAGGCA	257
	Rat: CACCCCTGGCATCTTCTCCTT	CATCCCAGCCTCCGTTATCCT	141

Caspase-3	Mouse: CGGGGTACGGAGCTGGACTGT	ATGCTGCAAAGGGACTGGATG	176
	Rat: GTCAGTCAGA GCGTAAGGAA	CAGTGCTCACAAGGTGGGTC	130

GAPDH	Mouse: CCACTCTTCCACCTTCGATG	TCCACCACCCTGTTGCTGTA	120
	Rat: AGTCCATGCC ATCACTGCCA	CATGTCAGATCCACAACGGA	300

Bax: Bcl2-associated X protein; Bcl-2: B-cell leukemia/lymphoma 2; GAPDH: glyceraldehyde-3- phosphate dehydrogenase.
